# Peer-Based Social Media Features in Behavior Change Interventions: Systematic Review

**DOI:** 10.2196/jmir.8342

**Published:** 2018-02-22

**Authors:** Sheik Mohammad Roushdat Ally Elaheebocus, Mark Weal, Leanne Morrison, Lucy Yardley

**Affiliations:** ^1^ School of Electronics and Computer Science University of Southampton Southampton United Kingdom; ^2^ Department of Digital Technologies Faculty of Information, Communication and Digital Technologies University of Mauritius Reduit Mauritius; ^3^ Academic Unit of Psychology Faculty of Social, Human, and Mathematical Sciences University of Southampton Southampton United Kingdom

**Keywords:** systematic review, social media, behavior control, health behavior, behavioral medicine, eHealth

## Abstract

**Background:**

Incorporating social media features into digital behavior change interventions (DBCIs) has the potential to contribute positively to their success. However, the lack of clear design principles to describe and guide the use of these features in behavioral interventions limits cross-study comparisons of their uses and effects.

**Objective:**

The aim of this study was to provide a systematic review of DBCIs targeting modifiable behavioral risk factors that have included social media features as part of their intervention infrastructure. A taxonomy of social media features is presented to inform the development, description, and evaluation of behavioral interventions.

**Methods:**

Search terms were used in 8 databases to identify DBCIs that incorporated social media features and targeted tobacco smoking, diet and nutrition, physical activities, or alcohol consumption. The screening and review process was performed by 2 independent researchers.

**Results:**

A total of 5264 articles were screened, and 143 articles describing a total of 134 studies were retained for full review. The majority of studies (70%) reported positive outcomes, followed by 28% finding no effects with regard to their respective objectives and hypothesis, and 2% of the studies found that their interventions had negative outcomes. Few studies reported on the association between the inclusion of social media features and intervention effect. A taxonomy of social media features used in behavioral interventions has been presented with 36 social media features organized under 7 high-level categories. The taxonomy has been used to guide the analysis of this review.

**Conclusions:**

Although social media features are commonly included in DBCIs, there is an acute lack of information with respect to their effect on outcomes and a lack of clear guidance to inform the selection process based on the features’ suitability for the different behaviors. The proposed taxonomy along with the set of recommendations included in this review will support future research aimed at isolating and reporting the effects of social media features on DBCIs, cross-study comparisons, and evaluations.

## Introduction

Supporting positive change in health behavior is a widely explored and active area of research commonly referred to as behavior change intervention (BCI) [1]. With advances in information and communication technologies (ICTs), researchers have developed digital behavior change interventions (DBCIs) [2], in which digital platforms such as the Web are used to deliver interventions targeting individuals whose physical locations may be different from that of the intervention provider, thus expanding the reach of the intervention. Along with this, DBCI presents other benefits such as reducing the financial and human resources usually required as input at such scale and enabling participants to engage with the interventions at times of their own choosing.

### Social Media in Digital Behavior Change Interventions

Using technologies such as the Web and sensor-rich phones potentially brings a tremendous leap toward large-scale BCIs. However, although many interventions focus on working at the individual participant level, an enormous amount of information that individuals share naturally with one another along with the accompanying peer support exchanged are left untapped. The use of social media features may provide new mechanisms to better understand individuals’ context and behaviors. These Web-based features enable users of an intervention to communicate or share data in virtual communities. A few examples of social media features are as follows: user profiles, groups, polls, online forums, etc. The features can help enhance the overall effectiveness of DBCIs by encouraging social interactions within interventions, promoting social support, and facilitating the adoption of social norm approaches. Social media features have been shown to be beneficial within intervention as identified in previous research in terms of increased motivation level and engagement with the interventions, for example [3-5].

A recent systematic review, which examined the use of online social networks (OSNs) in health BCIs, identified 10 research studies matching their set of criteria [6]. The use of social media features in DBCIs is an area of research that requires further examination. Two reviews exist that applied different search criteria, which did not seek to capture the full range of social media features included within DBCIs, including online forums, chat rooms, blogs, etc, which are not always defined within OSNs [7,8]. Systematic reviews with regard to the use of social media features in DBCIs that have been published tend to target only 1 [9,10] or 2 [11] out of the 4 behavioral risk factors published by the World Health Organization (WHO) as leading risk factors for global disease burden, which included tobacco smoking, alcohol use, physical inactivity, and diet [12]. This makes it hard to facilitate comparison across behaviors.

### Systematic Review of Social Media Inclusion

This review systematically identifies and analyzes peer-reviewed publications of DBCIs that include social media features or OSNs and target tobacco smoking, diet and nutrition, physical activities, or alcohol consumption. This creates an opportunity to have a better understanding about their effectiveness and how this differs for the various targeted behaviors. Although a taxonomy for the reporting of BCIs that focuses on standardizing definitions of techniques included in them was published [13], a corresponding taxonomy would be beneficial for social media features. Specifically, this paper presents the construction of a taxonomy of social media features, which will help in analysis and also provide guidance for selecting and including these features in interventions targeting specific risk factors. The behavioral outcomes in terms of users’ engagement and perceptions with regard to the inclusion of social media features are presented and analyzed. The impact and effectiveness of each of these features are also reported. A set of recommendations based on this review’s findings has been included to help researchers who are planning to include social media features in their behavioral interventions.

## Methods

### Identification of Studies

Information sources included literature searches that were conducted in the following health-related and multidisciplinary databases to ensure both the technical and behavioral aspects of interventions could be captured: Web of Science, Scopus, Engineering village, Medline, ERIC, CINAHL, PsycINFO, ProQuest, and Cochrane. Combined variants of relevant terms from the social media and DBCI domains were used to build a search query (eg, common social media terms such as *Facebook* and *forum* combined with terms such as *online* and *Web* and target behaviors such as *diet* and *smoking*). After refinements by 2 independent researchers, the finalized search query (see Multimedia Appendix 1) was used to conduct the searches. The search was conducted on November 30, 2015, with a time range between the year 2000 and the search date.

### Screening Process

The search results were downloaded, combined, and sorted for an initial filtering to remove duplicates. Then, 2 independent reviewers went through separate but identical copies of the result-set of unique entries to flag the nonrelevant ones based on our inclusion and exclusion criteria by going through their titles and abstracts. Differences were then resolved through consultation. The same reviewers conducted a subsequent eligibility screening of the remaining full-text articles.

To be included in the review, studies had to be (1) in the form of published and peer-reviewed full-text articles from conferences and journal papers and (2) targeting at least one of the following modifiable behavioral risk factors published by the WHO: “Tobacco use, physical inactivity, unhealthy diet and the harmful use of alcohol” [12].

No restrictions were placed on sample population used; participants from all age groups (including minors), gender, and health status were eligible for inclusion. Review papers for behavioral interventions that included references to studies matching our selection criteria were manually searched to identify studies that might have been missed in our initial search.

**Figure 1:**
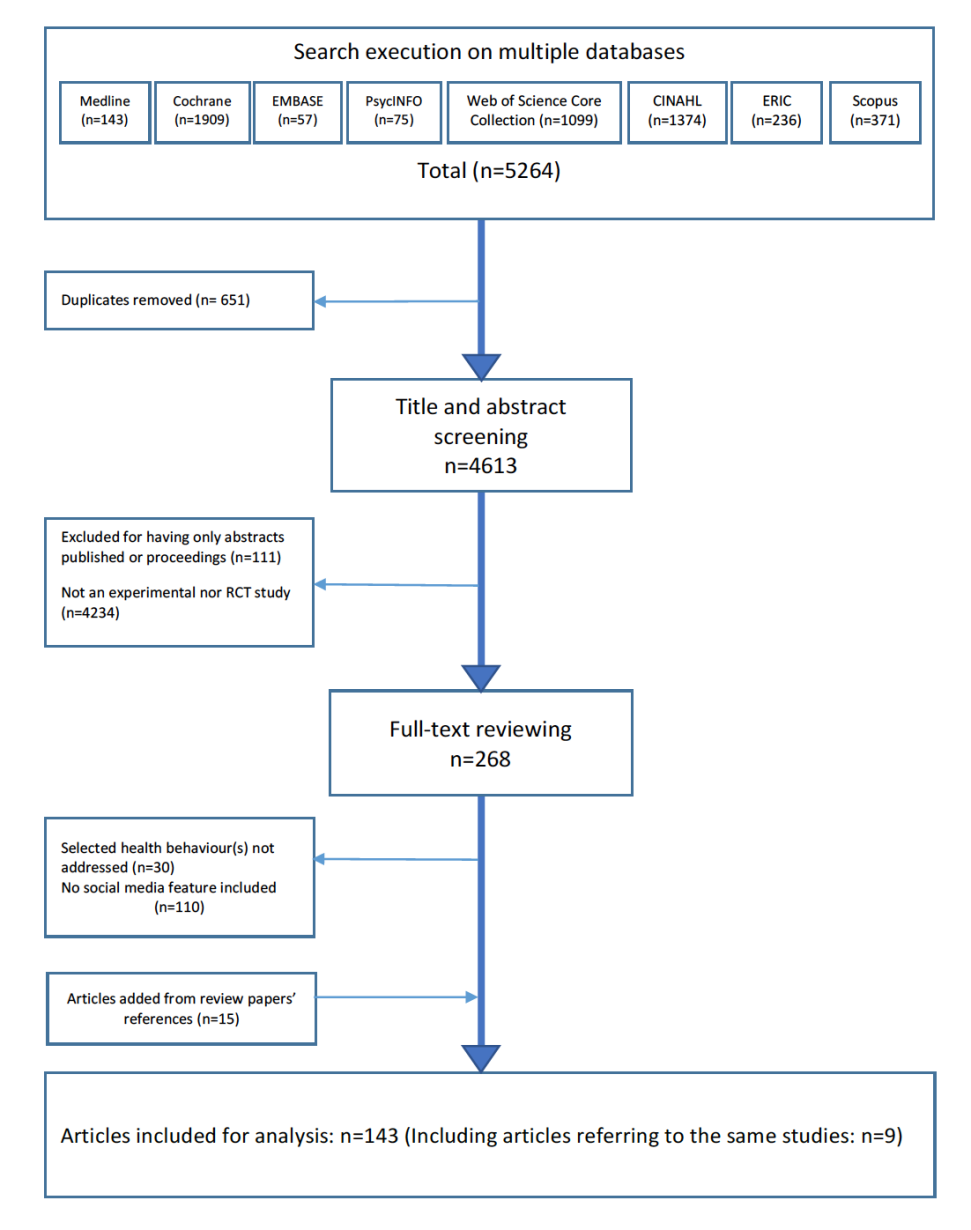
Flow diagram of the studies’ inclusion process.

A total of 143 publications were retained for data extraction and analysis as presented in Figure 1 according to the Preferred Reporting Items for Systematic Reviews and Meta-Analyses (PRISMA) guidelines. Out of these, 8 studies were reported through more than 1 publication (7 studies with 2 publications each and 1 study with 3 publications). Therefore, 134 studies were analyzed. Among these studies, 74 adopted a randomized controlled trials (RCT) design, with the remaining favoring a mostly experimental approach. However, they all provided valuable insights of social media features in their evaluation and were, therefore, included.

### Data Extraction and Analysis

A data extraction table was used to record key details for each study being reviewed (see Multimedia Appendix 2). Two reviewers were involved in this process. The first one performed the actual extraction, whereas the second reviewer verified the extracted data. The outcome of each of the studies was classified as positive, neutral, or negative. This classification was done by comparing the objective(s) and hypothesis stated for the studies with their reported results and findings. For studies that adopted an RCT design, when their intervention arm(s) were more effective than their control arm(s) by either or both the extent of change in behavior and the number of participants successfully adopting healthier behaviors, they were considered as having a positive outcome. In cases where no significant difference was reported between the intervention and control arms, the studies were considered as having a neutral outcome. Finally, for studies in which the control arm(s) were more effective in improving participants’ behavior than the intervention arm(s), the outcome was classified as negative. However, in the last situation, the reason(s) behind the control group outperforming the intervention arm would require further investigation, which is out of the scope of this review. Similarly, the same methods were adopted for studies with experimental or prepost designs by comparing the initial objective(s) and hypothesis with the studies’ findings.

The data table was analyzed for patterns of social media features’ inclusion in the different interventions to determine whether there was any correlation with the studies’ outcomes. The data were also used to inform the development of the taxonomy of social media features.

## Results

### Study Characteristics

A breakdown of the 134 studies reviewed, categorized by their targeted behaviors, is shown in Table 1. The majority of the studies targeted physical activity, either as a single behavior or in combination with other behaviors such as diet and nutrition. Alcohol consumption was addressed by the fewest number of studies.

### Taxonomy of Social Media Features

**Figure 2:**
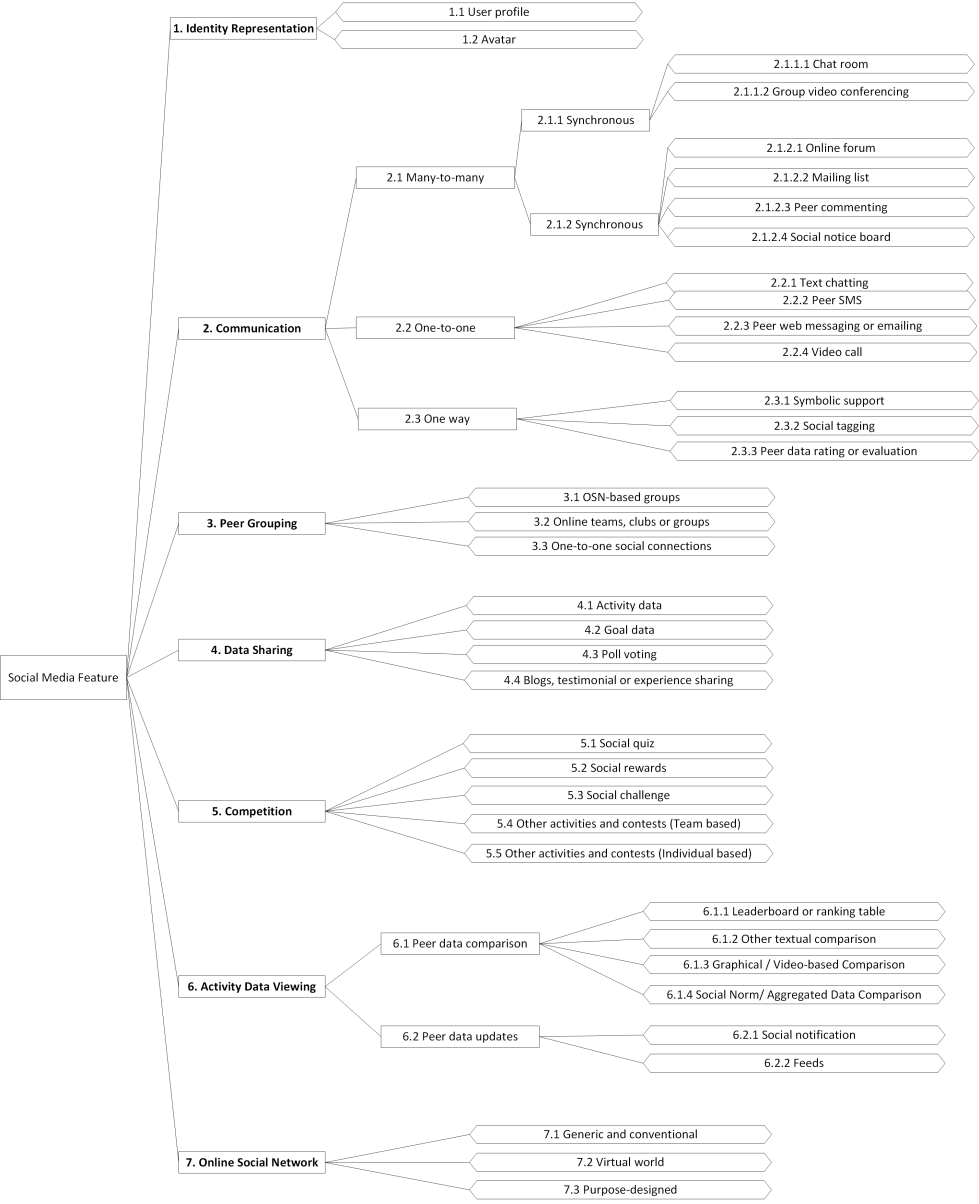
Taxonomy of social media features.

An initial prereview literature search was undertaken to identify potential social media features for incorporation in the search query for the review. The data extraction process for the publications reviewed led to the identification of an initial list of 29 social media features, with 70.1% (94/134) of the studies using more than 1 of these features. An initial set of hierarchical categories was then proposed and compared with one of the closest and related taxonomy published, which was by Michie et al [13]. This was undertaken by the main researcher and was reviewed by an independent researcher. Out of the 16 groups of techniques in Michie et al’s taxonomy, 6 of them were found to be relevant to social media features used in behavioral interventions, namely, goals and planning, feedback and monitoring, social support, comparison of behavior, reward and threat, and finally, identity. However, because of the fact that some social media features tended to be in multiple groups of techniques, a new hierarchical categorization better adapted for these features was proposed. This has been reviewed by 2 independent researchers to reach the final version presented in this paper. It is important to note that this taxonomy does not include an exhaustive list of social media features, but instead focused on those that are included in the 134 studies in this review. The list of social media features has been adapted to match the proposed taxonomy, which required in some cases the merging of 2 or more features (eg, blog and testimonial, and experience sharing) or the splitting of specific features into multiple ones (social challenge, contest, or competition). The final version of the taxonomy is presented in Figure 2.

Seven main categories of social media features have been identified, which are described in Table 2. A brief description of each social media feature in the taxonomy is provided in Multimedia Appendix 3.

**Table 1:** Breakdown of number of studies by addressed behaviors.

Addressed behaviors	n (%)
Alcohol consumption	5 (3.7)
Diet and nutrition	7 (5.2)
Diet and nutrition + physical activity	11 (8.2)
Diet and nutrition + physical activity + alcohol consumption	1 (0.7)
Physical activity	38 (28.4)
Physical activity + smoking cessation	1 (0.7)
Smoking cessation	25 (18.7)
Weight loss or weight maintenance + diet and nutrition	3 (2.2)
Weight loss or weight maintenance + diet and nutrition + physical activity	43 (32.1)
Total number of studies analyzed	134 (100.0)

**Table 2:** Categories of social media features.

Number	Category	Description
1	Identity representation	Used to provide information about an individual and his or her activities to peers and are usually customizable by the participant. This is usually either in the form of user profiles or avatars
2	Communication	Enable intervention participants to communicate with one another and could be further categorized as many to many (eg, chat rooms), one to one (eg, peer emailing), and one way (eg, thumbs up or likes)
3	Peer grouping	Grouping of participants based on characteristics such as age, geographical locations, or part of the same intervention arm, while ensuring that they are aware about others in their group and with the possibility to have some form of direct or indirect communication. Groups can consist of a minimum of 2 participants or^a^ OSN-based and non-OSN–based groups with more than 2 individuals
4	Data sharing	Enable participants of an intervention to share data about their activity, goals, or experience to either or both other participants and nonparticipants
5	Competition	Designed to introduce a competitive aspect in interventions through the use of features that enable participants to feel motivated while competing against one another (eg, social quiz)
6	Activity data viewing	Provide access to activity data of peers to participants through either regular updates (feeds and notifications) on a timely basis or enable them to compare their own data with that of their peers (eg, leaderboards)
7	Online social network (OSN)	The use of an Internet-based platform for enabling social interaction among intervention participants. OSNs can be subcategorized as either Generic and Conventional type (Facebook, mySpace, Twitter), Virtual World (SecondLife), or Purpose Designed (Yahoo Diet Diary, iWell, QuitNet, other intervention-specific proprietary OSNs). OSNs although being considered as a social media feature, usually act as a container for multiple other social media features

^a^OSN: online social network.

An important aspect to bear in mind when working with this taxonomy is that the different categories and their subcategories are not mutually exclusive and social media features are classified according to their most predominant properties. An example of an overlap between data sharing and peer data updates, where both are about the sharing of data, is that, for the latter, a user would receive updates about other peers without the peers actively involved in the sharing process (shared in an automated fashion), whereas, for the former, the peers would be actively sharing specific data with specific individuals or groups and could possibly have more fine-grained control over the sharing process.

### Social Media Features’ Inclusion

Communication-based features were the most prevalent and were included in a majority of studies, as shown in Table 3. Features from the competition, OSN, and identity representation categories were the least prevalent. The other high-level categories were peer grouping, activity data viewing, and data sharing. The inclusion of social media features from the different categories does not appear to differ by specific behaviors targeted. The only anomaly with this trend was for alcohol consumption. Studies with combinations of multiple behaviors were considered for each behavior individually.

The inclusion of specific social media features in studies targeting the different behaviors is presented in Multimedia Appendix 4.

When considering which addressed behaviors had the highest inclusion rate for each social media feature, physical activity and smoking cessation each had 11 highest inclusion rate instances, followed by dieting or nutrition and alcohol consumption with 5 instances each, and finally, 3 instances for weight loss or maintenance. The set of social media features that were the most included ones remained unchanged across all the behaviors considered in this review, except for alcohol consumption. These were online forums, social connections, and user profiles.

Social media features under the communication category remained the most popularly included across all the 5 behaviors considered in this review. Most of the studies included at least one social media feature based on communication, and with a consistent inclusion rate, 6 out of 10 studies in this review have made use of online forums as part of their interventions.

Features from the identity representation category and peer grouping category were more prevalent in interventions that addressed smoking cessation compared with other behaviors, with none of the interventions that targeted alcohol consumption using any of the features from this category.

Physical-activity related interventions were the most likely to include social media features from the competition category through social rewards. However, the highest inclusion rate from this category was below 12%. Although none of the interventions that addressed alcohol consumption included competition-based features, 2 smoking cessation interventions used features from this category, which included social quiz or activities and contests (individual-based) features.

**Table 3:** Studies that included social media features from the different categories.

SMF^a^ categories	Studies’ references
Communication	[3,5,14-141]
Peer grouping	[3,5,7,8,14,15,19,26,29-32,34,35,38,46,49,50,55,57,58,60,66,67,69,73,76,80,82,83,85,87,89,91,92,97,104-106,110,112, 113,118,121,124,126,127,129,130,133,139-147]
Data sharing	[3,14,19,26,31,32,34,38,41-43,46,49,54-57,59,60,72,73,76,80,81,85,92,97,103-106,110,112,118-120,126,127,133,138,146,148-150]
Competition	[3,5,7,8,15,19,38,39,42,46,49,50,54,57,67,87,91,98,104,109,112,118,133,142,151,152]
Activity data viewing	[3,5,7,8,14,19,26,27,29-32,34,38,39,46,49,50,54,55,57,58,60,66,67,73,76,80-83,85,87,91,92,97,103-105,126,127,129,130,133, 142-145,147-149,151,152]
Online social network	[3,5,14,15,19,26,29-32,35,46,49,55,57,58,60,67,69,76,80,83,85,91,92,97,104,105,114,118,126,129,130,133,138,140,141,143-145]
Identity representation	[3,5,14,15,19,26,29-32,35,38,40,46,49,50,54-58,60,67,76,80,82,83,85,91,92,97,98,104,105,126,129,130,134,140,141]

^a^SMF: social media features.

**Table 4:** Interventions reported outcomes while including social media features from the different categories.

Study outcome	Communication, n (%)	Peer grouping, n (%)	Data sharing, n (%)	Competition, n (%)	Activity data viewing, n (%)	Online social network, n (%)	Identity representation, n (%)
Studies with positive outcome	88 (71.0)	47 (84)	40 (93)	20 (77)	40 (80)	31 (84)	34 (87)
Studies with neutral outcome	33 (26.6)	9 (16)	2 (5)	6 (23)	10 (20)	6 (16)	5 (13)
Studies with negative outcome	3 (2.4)	0 (0)	1 (2)	0 (0)	0 (0)	0 (0)	0 (0)

For the peer data comparison subcategory of activity data viewing, interventions addressing physical activity were the most likely to include these features compared with other behaviors, with only 1 study in alcohol consumption and another from smoking cessation including a feature from this category.

OSNs of generic and conventional types were most popular in studies addressing weight loss or weight maintenance, followed closely by diet and nutrition and physical activity interventions. On the other hand, virtual worlds were included the most in interventions addressing smoking cessation. Although there was not much difference in the inclusion rate for purpose-designed OSNs for the 4 behaviors, studies that targeted smoking cessation used them the most.

### Social Media Features and Behavioral Outcomes

The majority of studies were classified as having positive outcomes (94/134, 70%), followed by neutral outcomes (37/134, 28%) and negative outcomes (3/134, 2%). Table 4 presents an analysis of the prevalence of social features by study outcome.

The majority of studies that included social media features reported positive outcomes, with the lowest percentage being 71% for the communication category and the highest at 93% for data sharing. Out of the 134 studies reviewed, only 4 studies reported negative outcomes. This trend persisted consistently across all the 5 behaviors considered, as shown in Table 5.

Studies that included social media features from the data sharing category were more likely to report positive outcomes for all the behaviors considered as compared with features from other categories. For example, one intervention that addressed physical activity, diet and nutrition, and weight loss or weight maintenance enabled participants to use blogs to share their personal experience [26]. In line with this, testimonial sharing was included in several studies addressing smoking cessation [14,31,32,55], which encouraged participants to share their own experience with others. Most of these studies also enabled the sharing of quit-smoking goals among one another. Although in most studies, data sharing through the variety of features under this category was initiated by participants, at least one study [34] addressing physical activity provided a functionality for participants to request others to share their data, which in this case was step counts. Haines-Saah et al [58], who used an OSN-based private group (peer-grouping category) for enabling participants to post photos (Data sharing category), reported gender bias in terms of engagement, whereby female participants tended to share more pictures and remained engaged for a longer period of time.

This was followed by identity representation and OSNs, both of which had significant overlaps due to the fact that OSNs were often used as a container for other social media features, with identity representation consisting of a user profile and avatar often being included. The most prevalent OSNs were QuitNet for studies addressing smoking cessation and Facebook for other behaviors. Studies that included features from the communication category reported higher levels of neutral and negative outcomes across the different behaviors compared with the other categories. Except for features from the communication category, studies addressing smoking cessation all reported positive outcomes (100%) for the other categories.

**Table 5:** Studies addressing different behaviors that reported positive, neutral, or negative outcomes.

Studies and their reported outcomes		Communication, n (%)	Peer grouping, n (%)	Data sharing, n (%)	Competition, n (%)	Activity data viewing, n (%)	Online social network, n (%)	Identity Representation, n (%)
**Physical activity**								
	Positive	57 (66)	31 (79)	^a^*24 (89)*	17 (74)	28 (76)	19 (76)	22 (81)
	Neutral	26 (30)	8 (21)	2 (7)	6 (26)	9 (24)	6 (24)	5 (19)
	Negative	3 (3)	0 (0)	1 (4)	0 (0)	0 (0)	0 (0)	0 (0)
**Diet and nutrition**								
	Positive	44 (71)	19 (83)	*17 (94)*	7 (70)	15 (75)	17 (89)	16 (89)
	Neutral	16 (26)	4 (17)	1 (6)	3 (30)	5 (25)	2 (11)	2 (11)
	Negative	2 (3)	0 (0)	0 (0)	0 (0)	0 (0)	0 (0)	0 (0)
**Smoking cessation**								
	Positive	21 (81)	*12 (100)*	*11 (100)*	*2 (100)*	*7 (100)*	*8 (100)*	*9 (100)*
	Neutral	5 (19)	0 (0)	0 (0)	0 (0)	0 (0)	0 (0)	0 (0)
	Negative	0 (0)	0 (0)	0 (0)	0 (0)	0 (0)	0 (0)	0 (0)
**Alcohol consumption**								
	Positive	4 (80)	*1 (100)*	*1 (100)*	*0 (0)*	*1 (100)*	0 (0)	0 (0)
	Neutral	1 (20)	0 (0)	0 (0)	0 (0)	0 (0)	0 (0)	0 (0)
	Negative	0 (0)	0 (0)	0 (0)	0 (0)	0 (0)	0 (0)	0 (0)
**Weight loss or weight maintenance**								
	Positive	33 (73)	12 (80)	*11 (92)*	5 (83)	9 (82)	11 (85)	10 (83)
	Neutral	10 (22)	3 (20)	1 (8)	1 (17)	2 (18)	2 (15)	2 (17)
	Negative	2 (4)	0 (0)	0 (0)	0 (0)	0 (0)	0 (0)	0 (0)

^a^Cells with the highest positive values have been indicated in italics.

#### Reported Impact of Social Media Features

Of the studies reviewed, 72.4% (97/134) published additional information about social media features that were included within their interventions either or both in their results and discussions sections. However, despite the high percentage, the type and amount of information provided about the social media features varied widely from one study to another, ranging from usage data (eg, frequency a feature was used, number of participants using it), impact-related information (eg, whether the feature had an effect on usage or behavioral outcomes), and participants’ perceptions (eg, usefulness, satisfaction, helpfulness, and social support derived). Similarly, the level of details varied from one descriptive sentence (eg, the studies by Block et al and Sternfeld et al [21,22]) to a full paragraph of text providing each social media feature’s statistical data and accompanying description (eg, the intervention by Napolitano et al [105]).

None of the studies that explicitly reported on the social media features described any negative impact on the outcome of the interventions. Indeed, 69.1% of the studies described the outcome of these features in positive terms and reported a range of effects attributed to their usage, such as higher levels of engagement with the interventions, increased perception of usefulness and satisfaction, as well as improvements in addressed behaviors attributed directly to the use of social media features. The remaining studies (n=30) reported either or both low usage (<50% of participants using a social media feature) and neutral outcomes.

#### Usage

The inclusion of social media features has been reported to be associated with increased user engagement in behavioral interventions.

##### Communication

Asynchronous features from the communication category (eg, online forums) were reported to support increases in usage and engagement [52,79,109,117,119,120], whereas studies that included synchronous features (eg, online video and chat rooms) for meetings [16,41,62-65] mostly reported no effect or reduced engagement when compared with controls (face-to-face). Interestingly, it was reported that female participants tended to engage in online group discussions more than their male counterparts [69]. Online forums have also been found to encourage usage over longer periods [109].

##### Peer Grouping and Data Sharing

Engagement was also found to be gender-biased in an OSN-based private group where participants could post photos, with females sharing more pictures and remaining engaged for longer periods of time [58]. Social connections were found to contribute toward motivating participants to engage more with the interventions [3,7,50,146]. Similar findings were also reported for peer-led support, leading to an increase in the frequency of participants visiting an intervention’s website [108].

Along with peer grouping–based features, interventions commonly included data sharing features [50,58,60]. The inclusion of polls was found to promote engagement the most as compared with other types of textual or graphical data [60], whereas the ability to create social connections to share each other’s activity data [50] or the use of an OSN-based private group to share photos [58] also produced high levels of engagement among their participants.

##### Competition and Activity Data Viewing

Competitive elements such as social challenges in interventions addressing physical activity were also found to promote engagement [7]. Linked with the competitiveness, the use of Leader Boards or Ranking tables from the Activity Data Viewing category caused participants who were interested with their ranking to access the intervention’s application more often [152]. Similarly, the inclusion of graphical-based comparisons [50] led to increases in user engagement.

##### Online Social Networks

It was reported that participants spent more time using an intervention that included an OSN platform [94]. Indeed, higher levels of engagements from participants were observed when OSNs were included along with their accompanying social media features [26,92]. However, this had no effect on attrition or retention rates [26,60].

#### Participants’ Perceptions: Social Support, Helpfulness, Satisfaction, and Motivation

Social media features are usually associated with enhanced social support and motivation perceived by participants.

Most of these studies included features from the communication category such as online forums and chat rooms. Although some of those that included online forums reported positive perceptions of social support among participants [40,72,98], there was also the possibility of no change [114] and even a demotivating effect based on content quality shared by participants [84,114]. Forums were often found to be useful or helpful by a majority of participants [15,27,40,51], although in some studies this dropped to below 50% of participants [42,93,95]. The lower percentages were attributed to a “lack of critical mass” in the number of participants engaging actively in the forums to change others’ perception positively [93] or could be based on participants’ preferring personal email counseling compared with peer-to-peer support from online forums [86]. It was reported that participants actively sought social support from peers through chat rooms and derived positive perception of social support [68,98], but this perception was lower as compared with in-person group meetings [65]. Other features that were associated with positively enhancing participants’ perception were group video chatting [41], mailing list [68], peer commenting [66], peer emailing [98], peer SMS text messages (short message service, SMS) [106,113,127], text chatting (one-to-one) [83], and symbolic support [66].

Peer grouping features were also found to help improve social support perceptions. Participants with access to these features had higher levels of social support coping [38,72,87,106,113]. However, this could also be attributed to participants feeling pressured in meeting goals or enjoying recognition and encouragements from peers [34,38,104]. Similar to content quality affecting motivation, social connections could have a demotivating factor in cases where the social support originates from better-performing peers [73].

Data sharing and peer data viewing had positive effects on perception of social support and motivation, especially when peers provide feedback [34,56,72,87,104,106,146]. However, data sharing was not always attributed with enhanced social support, with reported mixed effects when support originated from nonparticipants (external supporters) and their inability in constructing motivating messages [127] or possible concerns from participants about the usefulness to peers for the data being shared [42,149]. A positive correlation between the level of social support and the activeness of participants in data-sharing activities for an intervention addressing diet and nutrition was reported [66].

The inclusion of competition-based features in interventions was more likely to have a positive effect on participants’ perception of social support motivation levels in an intervention that addressed physical activity as at least one of its behaviors addressed [50,98], but this was not always the case [104]. Although rarely used in addiction-related behaviors, a feature from the Competition category, in the form of a quit-smoking contest in an intervention addressing Smoking Cessation, was perceived as “somewhat valuable” and had a low usage rate (35.3%) [104].

The only study among the reviewed articles to report on the impact on social support associated with the inclusion of *OSN* in behavioral interventions found no change in social support perception [114]. However, OSNs were among the most-reported features for perceived usefulness [15,26,58,60,67]. Although at least two interventions [15,26] used purpose-designed OSNs, Facebook was used as the OSN in the other interventions [58,60,67]. This perception of usefulness could likely be also linked to the prevalent popularity of Facebook as a generic OSN platform among individual users. Another interesting finding was the increased credibility perception among participants when a virtual world–type OSN with a recreated classroom along with an instructor avatar was used in an intervention addressing diet and nutrition and physical activity [35].

#### Behavioral Outcome

A total of 29 studies reported on the effectiveness of some of the social media features included in their interventions in contributing to change participants’ behaviors (see Multimedia Appendix 5). These features were from 5 of high-level categories in our taxonomy, namely, identity representation, communication, peer grouping, activity data viewing, and OSN.

Communication-based social media features included in behavioral interventions were among the most reported for their effectiveness in modifying behaviors. Interactions among participants through asynchronous features such as forums were reported to have led to behavior change in at least four studies [75,79,108,135,136] that addressed a combination of physical activity, diet and nutrition, and weight loss. However, in at least one study, this change was minimal and only found among female participants [75]. Forums were among the most reported in studies addressing smoking cessation with regard to its effect in changing participants’ behavior positively [31,32], although in at least one case no effect was found [117]. Chat rooms, enabling synchronous communication among participants were also reported to effectively modify behavior in some studies. Among interventions that addressed physical activity, diet and nutrition, and weight loss or weight maintenance, chat rooms were found to be effective in 2 interventions [76,135,136], whereas no effects were observed in others [62-64]. Smoking Cessation interventions could also benefit from their effectiveness, as it has been reported that participants with access to chat rooms were more likely to report abstaining from smoking [141]. One-to-one communication-based features have been reported to increase abstinence rates [14,55], increase physical activity, and lead to weight loss [57].

The use of peer grouping–based features was found to result in a number of positive outcomes, such as weight loss among studies addressing weight loss or weight maintenance, diet and nutrition, and physical activity [5,8,76,118,129], increase in activity levels for studies that addressed only physical activity [50,73,81,82], and also increase in the likeliness of quitting smoking in smoking cessation interventions [31,38,55].

*Activity data viewing* was also reported to have a positive impact on behavior change among studies addressing physical activity. Social interactions through games and allowing viewing of peers’ performance [46,133] led to increases in physical activity levels.

*OSNs* used as part of behavioral interventions were also associated with positive behavior change, especially for weight loss [57,76,118,143-145], although this was not always the case [26]. All these studies addressed multiple behaviors, namely, physical activity, diet and nutrition, and weight loss or weight maintenance. Improvements in dietary awareness were also attributed to the use of OSNs [85]. OSNs that included user profiles were also found to encourage participants to smoke less and cause an increase in intentions to quit [140,141].

## Discussion

### Principal Findings

This review found that the majority of studies targeted either physical activity or a combination of behaviors that included physical activity (eg, physical activity, diet and nutrition, and weight loss or weight maintenance). The use of a specific social media feature in interventions addressing such combinations could be potentially risky in certain circumstances, such as a specific social media feature being found to highly encourage an individual to improve a particular behavioral aspect when that behavior is targeted as a single one, but in an intervention combining a second behavior, its effectiveness might be nullified or even create an opposite outcome. This review has not analyzed this aspect in more detail. However, it would be desirable to investigate these effects further.

Physical activity and smoking cessation interventions had the highest prevalence of social media features. Among these features, however, some were consistently more popularly included than others across all the different behaviors considered. Most of the behavioral interventions that included social media features reported positive (>70%) outcomes with respect to their set of objectives and hypothesis. Interventions that included social media features from the data sharing category had the highest positive outcome percentages (>88.9%). The main effects identified to be associated with the inclusion of social media features in behavioral interventions were about usage or engagement of participants; enhanced perception of social support, helpfulness, satisfaction, and motivation; and lastly, behavioral outcome.

It is possible that social media features were found to be more prevalent in physical activity interventions because features that draw on social behavior change techniques are more relevant to this behavior. This is in line with and adds to the findings of McCully et al’s [153] survey that reported an increasing viability for using the Internet as a platform for delivering behavioral interventions on large scales. However, it should be pointed out that in most of the reviewed studies, their outcomes were not always explicitly attributed to the impact of the social media features that were included as was also reported in Chang et al’s review [9].

The studies reviewed used nonstandard ways of reporting on the social media features by using different names to refer to the same feature and with varying levels of details. For example, online forums were also referred to as messaging board, bulletin board, discussion forum, discussion board, etc. In terms of description, these forums were sometimes moderated by intervention counselors, but not all studies described whether these features were moderated or the extent that counselors were involved in the group discussions. Some studies made use of generic online social networking sites such as Facebook and QuitNet, whereby a large number social features were available to the intervention participants without the researchers necessarily describing them; these features were however included in our analysis. These issues closely relate to the justifications put forward by Michie et al [13] that led to the proposal of a taxonomy for the reporting of BCIs. Without detailed and standardized description, it is not possible to draw comparisons across different studies on the use and impact of social media features. The taxonomy of social media features proposed from this review can support future research by informing more standardized and detailed descriptions that will facilitate cross-study comparison.

Our review identified that similar social media features (eg, online forums) were associated with positive outcomes across different behaviors. This suggests that although mostly prevalent in physical activity interventions, social media features might be relevant for a variety of health issues. However, this could also be the result of intervention designers including these features without enough consideration about their suitability and effectiveness on users for the different types of behaviors addressed and could, instead, be more focused on maximizing functionalities in their interventions. Therefore, there is a strong justification to empirically test the suitability and impact of including social media features in behavioral interventions.

Three main areas were identified with regard to the effects of social media features on users, namely, usage, participants’ perception, and behavioral outcome. These effects may be attributed to the social influence element of these features reported in previous research [3,4] that found a positive impact in sustaining behavior change. A number of studies reported that the inclusion of social media features increased user engagement in behavioral interventions, and in at least one study, higher levels of sustained user engagement through interaction with multiple social media features was reported [49]. However, when compared with face-to-face alternatives, social media features were found to produce lower levels of engagement. Among the studies that reported the perceived usefulness or helpfulness, or sense of enjoyment and satisfaction of participants when using social media features included in interventions, more than half referred to features from the communication category, more specifically, online forums. Interestingly, in most cases, participants with access to these features felt pressured, motivated, or felt both to achieve goals, contrary to the findings of Dennison et al [154] that reported some degree of reluctance and feeling of embarrassment when participants’ data were shared among peers. However, social support originating from better-performing peers [73] or in the form of poor support content quality from peers [84,114] did have a demotivating effect in a few studies reviewed. Although often assumed by researchers that social media features could result in lowering of attrition rate, this effect was minimal, with only one study [109] reporting an increase in likeliness for participants to complete an intervention, whereas 2 other studies reported finding no such effect [26,60]. In terms of social media features affecting behavioral outcome, our analysis found that the most effective features were communication-based, and more specifically, asynchronous ones (eg, online forums). Indeed, features from this subcategory of communication are known to provide more direct social support either from peers or trained professionals interacting with users on a one-to-one or one-to-many basis. This in turn can have an impact on behavioral outcomes [155,156].

The low level of focus on privacy based on the limited amount of information provided by the studies reviewed regarding this aspect associated with the sharing and peer viewing of participants’ data is an area that requires further attention. Although privacy might not have been an issue where the intervention’s data were accessible only to their respective users and their therapists, social media features unlock the potential of data being shared among peers. Another potential challenge of including social media features was the associated cost for moderating shared data, but this was explicitly mentioned by only 1 study [125], which reported that moderation of the online discussion groups (online forums) was the most significant cost of their study.

### Limitations and Strengths of the Review

Among the reviewed studies, there was a lack of information reported on the social media features included and their impact on whether the behaviors addressed were affected directly or indirectly by them. This was in line with Michie et al [13], who found that BCIs with poor descriptions in their research protocols and study reports made them challenging to evaluate their effectiveness and to replicate.

This review included studies from the year 2000 as social media really started gaining popularity from around then. Although it is a fact that there have been a lot of new technologies and changes in the field of ICT, including social media, most basic concepts are still being used, although often adapted to match the current level of technology. Therefore, the social media features included in those studies are still relevant. Accordingly, diligent care must be taken when interpreting this review’s findings in the current context. For example, results from the use of social media in recent years would be different from that of the previous decade because of the ways that they are used through different types of devices and interfaces. With our taxonomy, however, future extensions will be possible and help researchers to analyze and compare the evolvement of social media features in behavioral interventions.

This work has adopted a systematic approach for reviewing behavioral interventions, which included social media features across a wide range of behaviors, which has the potential to be used as a foundation for future research in the area. Along with the review, a taxonomy for categorizing social media features has also been presented. The taxonomy does not consist of an exhaustive list of social media features but rather focuses on those that were included in the reviewed studies.

### Future Work

As this is the first attempt at producing a taxonomy for social media features included in behavioral interventions, it is expected that continued refinement will be carried out for standardizing the names and descriptions of the different categories and features in concert with researchers from both Social Science and Computer Science. More research needs to be carried out to find ways for isolating the effects of social media features on intervention users, as this review has found that many studies do not report on these aspects clearly. The complete dataset of studies reviewed consisting of their different attributes such as social media features used, sample size, and behavior(s) addressed has been included in Multimedia Appendix 2.

### Recommendations

This review has found that although social media features are being widely included in behavioral interventions, little research-based evidence of their effectiveness in modifying behaviors exist. There appears to be tendency to use these features based on convenience and popularity rather than their suitability for specific behaviors. We are, therefore, proposing a set of recommendations aimed at researchers and intervention designers to help evaluate and maximize the effectiveness of social media features on behavioral intervention participants.

First, develop and use a uniform and well-defined labeling scheme for social media features with the help of the taxonomy presented in this review. This will greatly facilitate future research work attempting to identify social media features included in behavioral interventions for comparison and reviewing.

Second, better design of studies capable of isolating, describing, and evaluating the impact of including social media features on participants and on the interventions’ overall outcomes, both qualitatively and quantitatively, while ensuring the following aspects are covered: impact of social media features included in behavioral interventions on usage, perception of social support, helpfulness and satisfaction, attrition, and credibility. As reported in this review, the positive or negative impact for the different studies included was quite subjective, and therefore, they could only be cross-evaluated superficially. Should future studies publish sufficient quantitative data as part of their results, effects size could then be used as a selection criteria for systematic reviews.

Third, conduct more research focused on user’s experience of social media features and factors such as privacy and cost in their use to understand how they can best be implemented. A user-centered design approach aimed at ensuring that any concerns from the users’ point of view are taken on board from an early stage could be considered. As a result, this could potentially increase users’ engagement with the social media features.

### Conclusions

It was found that a majority of studies in this review reported positive outcomes with respect to their objectives and hypothesis. A new taxonomy of social media features used in behavioral interventions has been developed that will support researchers and intervention designers in comparing social media features and guiding their future inclusion in behavioral interventions with better consistency. Social media features were reported to increase usage; enhance perceived levels of social support, motivation, and feeling of satisfaction; and also having a direct effect in behavioral outcome. The main concerns identified with respect to the inclusion of social media features in behavioral interventions were, first, an underreported methodical selection process based on their suitability for specific behaviors and other contextual elements. Another issue uncovered was the nonstandardized way to identify and describe social media features and their effects on intervention users. Moreover, little information has been published with respect to the privacy and cost issues associated with social media features’ inclusion in behavioral interventions. Therefore, more research on these aspects has been recommended.

## Appendix

Multimedia Appendix 1 Search query construction.

Multimedia Appendix 2 Data table extracted from studies reviewed.

Multimedia Appendix 3 Social media features’ description.

Multimedia Appendix 4 Inclusion of specific social media features by behavior.

Multimedia Appendix 5 Social media features leading to behavioral change.

## Abbreviations

BCI: behavior change intervention
DBCIs: digital behavior change interventions
ICTs: information and communication technologies
OSN: online social network
PRISMA: Preferred Reporting Items for Systematic Reviews and Meta-Analyses
RCTs: randomized controlled trials
SMS: short message service
WHO: World Health Organization
