# Supplementary material for: Peer-Based Social Media Features in Behavior Change Interventions: Systematic Review
Source: J Med Internet Res. 2018 Feb 22;20(2):e20. doi: 10.2196/jmir.8342 (PMC5843796; doi:10.2196/jmir.8342)
Supplement: Multimedia Appendix 1 [file jmir_v20i2e20_app1.pdf]

1. Social Media	social network* OR social feature* OR social tool* OR social component* OR social media OR social support OR peer support OR facebook OR linkedin OR twitter OR badoo OR orkut OR myspace or youtube or Instagram OR poll* OR survey* OR questionnaire* OR group* OR messag* OR leader board* OR rank* table* OR profile OR forum OR quiz OR diary OR diaries OR knowledge repositor* OR progress viewing OR notification* OR rival nomination OR goal setting OR sharing OR comment* OR feed* OR reminder* OR self-reporting tool* OR planner* OR chat*
2. Internet	online OR web OR internet
3. Mobile	smartphone OR mobile OR android OR iphone
4. Intervention	behaviour change intervention* OR behavior change intervention* OR digital intervention*
5. Target Behaviours	(alcohol OR binge drink* OR healthy eating OR nutrition OR diet* OR , exercis* OR sport* OR sedentary* OR physical OR inactiv* OR motor activit* OR tobacco OR smoking OR nicotine OR weight loss)
6. Combined	1 AND (2 OR 3) AND 4 AND 5
Query:	(social network* OR social feature* OR social tool* OR social component* OR social media OR social support OR peer support OR facebook OR linkedin OR twitter OR badoo OR orkut OR myspace or youtube or Instagram OR poll* OR survey* OR questionnaire* OR group* OR messag* OR leader board* OR rank* table* OR profile OR forum OR quiz OR diary OR diaries OR knowledge repositor* OR progress viewing OR notification* OR rival nomination OR goal setting OR sharing OR comment* OR feed* OR reminder* OR self-reporting tool* OR planner* OR chat*)<br>AND<br>((online OR web OR internet)<br>OR<br>(smartphone OR mobile OR android OR iphone))<br>AND<br>(behaviour change intervention* OR behavior change intervention* OR digital intervention*)

	AND<br>(alcohol OR binge drink* OR healthy eating OR nutrition OR diet* OR , exercis* OR sport* OR sedentary* OR physical OR inactiv* OR motor activit* OR tobacco OR smoking OR nicotine OR weight loss)
