# Supplementary material for: Peer-Based Social Media Features in Behavior Change Interventions: Systematic Review
Source: J Med Internet Res. 2018 Feb 22;20(2):e20. doi: 10.2196/jmir.8342 (PMC5843796; doi:10.2196/jmir.8342)
Supplement: Multimedia Appendix 3 [file jmir_v20i2e20_app3.pdf]

Category/Sub-category	SOCIAL MEDIA FEATURE	DESCRIPTION
1. Identity Representation	1.1 User Profile	A digital representation of a participant with associated personal data which can be made viewable by other participants fully, partly or kept as private depending on interventions. Can include guest book for receiving comments, status, profile picture, avatar etc. Interventions using Online Social Networks, namely Facebook and QuitNet were assumed to have use profiles.
	1.2 Avatar	Avatars are graphical representation of participants and visible to one another.
2.1.1 Communication > Many to Many > Synchronous	2.1.1.1 Chatroom (Text-based)	Text-based synchronous group communication feature.
	2.1.1.2 Group Video Conferencing	Video-based synchronous group communication feature.
2.1.2 Communication > Many to Many > Asynchronous	2.1.2.1 Online forum	Web-based feature enabling participants to post and/or read one another's messages usually organised in the form of topics/threads.
	2.1.2.2 Mailing list	Electronic mail (email) used by intervention participants to send and receive messages to and from a group of participants in the mailing list.
	2.1.2.3 Peer commenting	Posting and reading of comments by intervention participants on posts, responses, blog entries, articles, etc. Includes Textual, Graphical, and Audio-based format. This excludes commenting on online forums. All studies using Facebook or QuitNet were assumed to have this feature.
	2.1.2.4 Social Notice Board (Group walls)	Area on an intervention's website or online group where participants can publish, read and comment on one another's posts.
2.2 Communication >One to One	2.2.1 Text chatting	Internet-based private messaging / chatting between two intervention participants in synchronous mode. All studies using Facebook were assumed to have this feature.
	2.2.2 Peer SMS	Phone-based text message/ Short Message Service (SMS) used by intervention participants to send messages to one another.
	2.2.3 Peer Web Messaging / Emailing	Electronic mail (email) or Internal messaging used by intervention participants to send messages to one another in Asynchronous mode. All studies using Facebook or QuitNet were assumed to have this feature.
	2.2.4 Video Call	Video-based synchronous communication feature for one-to-one video call among participants.
2.3 Communication > One-way	2.3.1 Symbolic Support	Support provided by participants to one another in the form of 'Thumb ups', 'Likes', 'Smile' and other related symbolic ways. All studies using Facebook or QuitNet were assumed to have this feature.
	2.3.2 Social Tagging	Participants viewing one another shared data can post tags (descriptive, categories, etc.).Tags posted or search terms used by participants can help generate tag clouds.
	2.3.3 Peer Data Rating / Evaluation	Participants providing ratings or evaluating one another's' shared data.
3. Peer Grouping	3.1 Online Teams/ clubs/ groups	Usually created by intervention designers to group participants into small teams/clubs to facilitate sharing and viewing of data among peers. Excludes all OSN-based groups.
	3.2 OSN-based groups	Groups created in online social networks usually by intervention designers and participants are either added by them or given the choice whether to join the groups. Participants in a group can share resources, photos, comments,etc.

	3.3 One-to-one social connections (friending/buddy nomination)	Functionalities enabling either participants to create virtual links between themselves and their peers or for researchers and therapists to create these links on the participants' behalf. All interventions making use of online social networks are assumed to include one-to-one social connections.
4. Data Sharing	4.1 Activity Data	Features enabling participants to either manually share their activity data or customising how their data is shared to others (what data, to whom, when, manual/automated, etc.).
	4.2 Goal Data	Sharing goal/planning data by participants to peers. All studies using QuitNet were assumed to have this feature.
	4.3 Poll Voting	Voting in polls by participants and thereby sharing one's own opinions and choices to other participants.
	4.4 Blogs /Testimonial/ Experience Sharing	Testimonial-based posts shared in specific areas (for example blogs, forums, notice boards, specially designated locations) in textual, video or audio format and viewable by other participants who can then respond. All studies using QuitNet were assumed to have this feature.
5. Competition	5.1 Social Quiz	Completing intervention related quizzes and comparing their results with other participants' aggregated results.
	5.2 Social Rewards	Virtual rewards (for example badges, trophies, points) received by a team or an individual participant for completing a challenge, winning a contest or as encouragement from friends and visible/announced to other participants.
	5.3 Social Challenge	Group-based challenges related to achieving behaviours targeted by interventions created by intervention experts or the participants themselves.
	5.4 Activities & Contests (Team-based)	Competitive activities or contests undertaken by a participant as part of a group/team against other group(s)/team(s).
	5.5 Activities & Contests (Individual-based)	Competitive activities or contests undertaken by a participant as an individual with the possibility to compare their performance with other participants.
6.1 Activity Data Viewing > Peer Data Comparison	6.1.1 Leaderboard / Ranking Table	A scoreboard or ranking table showing participants and their current scores usually sorted by highest achievers.
	6.1.2 Other Textual Comparison	Other form of data viewing in the form of text (for example, comments by participants describing their achievements) which enables a participant to compare other participants' activities / achievements with their own.
	6.1.3 Graphical/Video-based Comparison	Enabling the graphical/video based comparison of peers' data (for example, an image of a race track with icons/avatars representing peers) which enables a participant to compare other participants' activities /achievements with their own.
	6.1.4 Social Norm / Aggregated Data Comparison	Feature to enable a participant to compare his/her own performance/data with an average of all the participants in his/her group.
6.2 Activity Data Viewing > Peer Data Updates	6.2.1 Social Notification	Notification in the form of graphical icons or text-based description sent to intervention participants to inform them about activities, changes in status, etc. of other participants.
	6.2.2 Feeds	Frequently updated data for the different activities related to targeted behaviours undertaken by other participants formatted in such a way that a particular

		participant can follow them. All studies using Facebook or QuitNet were assumed to have this feature.
7. Online Social Network	7.1 Generic & Conventional	An Internet-based platform for enabling social interact among its members. Generic & Conventional OSNs are usually accessible to the general public and by design are not intended for use within interventions although they provide some functionality such as privacy settings to restrict resources. Examples include Facebook, Twitter, MySpace
	7.2 Virtual World	Three-dimensional version of online social networks such as SecondLife.
	7.3 Purpose-Designed	Online social networks designed for interventions addressing specific behaviours. Some of these OSNs are available as off-the-shelf applications while others are designed for specific interventions and are proprietary.
