# Supplementary material for: Peer-Based Social Media Features in Behavior Change Interventions: Systematic Review
Source: J Med Internet Res. 2018 Feb 22;20(2):e20. doi: 10.2196/jmir.8342 (PMC5843796; doi:10.2196/jmir.8342)
Supplement: Multimedia Appendix 4 [file jmir_v20i2e20_app4.pdf]

Multimedia Appendix 4. Inclusion of specific social media features by behavior.

Category or subcategory	[a]SMFs	All studies reviewed, n/N (%)	Physical activity, n/N (%)	Diet and nutrition, n/N (%)	Smoking cessation, n/N (%)	Alcohol consumption, n/N (%)	Weight loss or weight maintenance, n/N (%)
Identity representation	User profile	35/134 (26.1)	23/94 (24)	17/65 (26)	*9/26 (35)*[b]	0/6 (0)	11/46 (24)
	Avatar	9/134 (6.7)	7/94 (7)	3/65 (5)	*2/26 (8)*	0/6 (0)	2/46 (4)
	All SMFs under identity representation		27/94 (29)	18/65 (28)	*9/26 (35)*	0/6 (0)	12/46 (26)
Communication▶ many to many ▶ synchronous [c]	Chat room (text based)	22/134 (16.4)	13/94 (14)	14/65 (21)	6/26 (23)	*2/6 (33)*	11/46 (24)
	Group video conferencing	3/134 (2.2)	3/94 (3)	2/65 (3)	0/26 (0)	0/6 (0)	*2/46 (4)*
Communication▶ many to many ▶ asynchronous	Online forum	78/134 (58.2)	58/94 (62)	41/65 (62)	16/26 (62)	3/6 (50)	*32/46 (70)*
	Mailing list	5/134 (3.7)	3/94 (3)	3/65 (5)	1/26 (4)	*1/6 (17)*	2/46 (4)
	Peer commenting	33/134 (24.6)	21/94 (22)	16/65 (24)	*8/26 (31)*	0/6 (0)	10/46 (22)
	Social notice board (group walls)	18/134 (13.4)	12/94 (13)	*11/65 (17)*	2/26 (8)	0/6 (0)	7/46 (15)
Communication▶ one to one	Text chatting	19/134 (14.2)	13/94 (14)	9/65 (14)	2/26 (8)	*1/6 (17)*	6/46 (13)
	Peer SMS[d] text messages	7/134 (5.2)	3/94 (3)	2/65 (3)	4/26 (15)	*1/6 (17)*	1/46 (2)
	Peer web messaging or emailing	33/134 (24.6)	23/94 (24)	17/65 (26)	6/26 (23)	1/6 (17)	*14/46 (30)*
	Video call	1/134 (0.7)	1/94 (*1*)	0/65 (0)	0/26 (0)	0/6 (0)	0/46 (0)
Communication▶ one way	Symbolic support	22/134 (16.4)	12/94 (13)	10/65 (15)	*6/26 (23)*	0/6 (0)	6/46 (13)
	Social tagging	3/134 (2.2)	2/94 (2)	*2/65 (3)*	0/26 (0)	0/6 (0)	0/46 (0)
	Peer data rating or evaluation	1/134 (0.7)	0/94 (0)	*1/65 (2)*	0/26 (0)	0/6 (0)	0/46 (0)
	All SMFs under communication		86/94 (92)	61/65 (94)	*26/26 (100)*	5/6 (83)	44/46 (96)

Peer grouping	Online teams, clubs or groups	9/134 (6.7)	*9/94 (10)*	2/65 (3)	0/26 (0)	0/6 (0)	1/46 (2)
	OSN[e]-based groups	14/134 (10.4)	9/94 (10)	*7/65 (11)*	2/26 (8)	0/6 (0)	5/46 *(11)*
	One-to-one social connections (friending or buddy nomination)	50/134 (37.3)	33/94 (35)	22/65 (33)	*12/26 (46)*	1/6 (17)	14/46 (30)
	All SMFs under peer grouping		39/94 (41)	23/65 (35)	*12/26 (46)*	1/6 (17)	15/46 (33)
Data sharing	Activity data	21/134 (15.7)	*17/94 (18)*	6/65 (9)	3/26 (12)	0/6 (0)	4/46 (9)
	Goal data	6/134 (4.5)	1/94 (1)	0/65 (0)	*5/26 (19)*	0/6 (0)	0/46 (0)
	Poll voting	8/134 (6.0)	4/94 (4)	*6/65 (9)*	2/26 (8)	0/6 (0)	*4/46 (9)*
	Blogs, testimonial, or experience sharing	16/134 (11.9)	8/94 (9)	8/65 (12)	*6/26 (23)*	1/6 (17)	6/46 (13)
	All SMFs under data sharing		33/94 (35)	*24/65 (37)*	7/26 (27)	1/6 (17)	13/46 (28)
Competition	Social quiz	3/134 (2.2)	2/94 (2)	2/65 (3)	*1/26 (4)*	0/6 (0)	0/46 (0)
	Social rewards	11/134 (8.2)	*11/94 (12)*	4/65 (6)	0/26 (0)	0/6 (0)	3/46 (7)
	Social challenge	5/134 (3.7)	*5/94 (5)*	2/65 (3)	0/26 (0)	0/6 (0)	1/46 (2)
	Activities and contests (team based)	3/134 (2.2)	*3/94 (3)*	1/65 (2)	0/26 (0)	0/6 (0)	0/46 (0)
	Activities and contests (individual based)	12/134 (9.0)	*10/94 (11)*	5/65 (8)	1/26 (4)	0/6 (0)	4/46 (9)
	All SMFs under competition		*23/94 (24)*	10/65 (15)	2/26 (8)	0/6 (0)	6/46 (13)
Activity data viewing ▶ peer data comparison	Leaderboard or ranking table	8/134 (6.0)	*8/94 (9)*	2/65 (3)	0/26 (0)	0/6 (0)	1/46 (2)
	Other textual comparison	10/134 (7.5)	*9/94 (10)*	2/65 (3)	1/26 (4)	0/6 (0)	1/46 (2)
	Graphical or video-based comparison	7/134 (5.2)	*7/94 (7)*	3/65 (5)	0/26 (0)	0/6 (0)	2/46 (4)
	Social norm or aggregated data comparison	8/134 (6.0)	6/94 *(6)*	3/65 (5)	0/26 (0)	*1/6 (17)*	0/46 (0)

Activity data viewing ▶ peer data updates	Social notification	3/134 (2.2)	*2/94 (2)*	*1/65 (2)*	0/26 (0)	0/6 (0)	0/46 (0)
	Feeds	26/134 (19.4)	17/94 (18)	12/65 (18)	*6/26 (23)*	0/6 (0)	8/46 (17)
	All SMFs under activity data viewing		*37/94 (39)*	20 (31)	7/26 (27)	1/6 (17)	11/46 (24)
OSN	Generic and conventional	16/134 (11.9)	11/94 (12)	8/65 (12)	2/26 (8)	0/6 (0)	*6/46 (13)*
	Virtual world	17/134 (12.7)	12/94 (13)	9/65 (14)	*4/26 (15)*	0/6 (0)	6/46 (13)
	Purpose-designed	4/134 (3.0)	2/94 (2)	2/65 (3)	*2/26 (8)*	0/6 (0)	1/46 (2)
	All SMFs under OSN		25 (27)	19 (29)	*8/26 (31)*	0/6 (0)	13/46 (28)

[a]SMF: social media features.

[b]The features are grouped based on our taxonomy with the highest percentage for each category of social media features in italics.

[c]▶: symbol used to denote a subcategory.

[d]SMS: short message service.

[e]OSN: online social network.
