# Supplementary material for: Peer-Based Social Media Features in Behavior Change Interventions: Systematic Review
Source: J Med Internet Res. 2018 Feb 22;20(2):e20. doi: 10.2196/jmir.8342 (PMC5843796; doi:10.2196/jmir.8342)
Supplement: Multimedia Appendix 5 [file jmir_v20i2e20_app5.pdf]

Social media features leading to behavioral change

**Studies**		OSN	Communication (Many to Many >> Asynchronous)	Communication (Many to Many >> Synchronous)	Communication (One to One)	Peer Grouping	Activity Data Viewing	Identity Representation	
[26]	W+D+P	X							OSN capabilities site had no significant effect for weight loss.
[62-64]				X					Chatroom-based online meetings had no effect on the outcome (weight loss).
[75]			X						No link between use of online forums and weight loss among male but a small effect was found among female participants.
[79]			X						Guided discussion group in online forums led to significant reduction in weight/shape concerns compared to control.
[108]			X						Peer-led support in online forums found to contribute to weight loss.
[8]						X			Participants in teams (online groups) reported higher social influence for weight loss, led to higher percentage of weight loss.
[5]						X			Number of social connections/virtual friends: linked to weight loss and their friends weight loss (positive correlation).
[76]		X		X		X			Evidence that social support components (friends, virtual world, chatrooms) related to weight loss.
[57]		X			X				Participants using OSN features more, especially sending private messaging tended to increase time walking and lost more weight.
[143-145]		X							Engagement with twitter related to weight loss.
[135, 136]			X	X					Participants who attended more chat sessions achieved greater weight loss., number of posts to online forum was also associated with weight loss.
[118]	W+D	X				X			Higher social interaction on OSN associated with higher weight loss.
[129]		X				X			The use of Facebook groups was significantly related to weight loss at 3 and 6 months.
[85]	D+P	X						X	Significant improvements in dietary awareness but no difference in PA among participants with access to purpose-built OSN with user profiles but without direct peer-to-peer communication.
[133]	P						X		Significant increase in number of steps taken by participants during the social interaction and social game conditions compared to the control.
[81]						X			Increase in step counts for version with social component (viewing team progress).
[82]						X			Larger decreases among participants in social groups in terms of sedentary behaviour.
[46]			X				X		Higher physical activity in social condition compared to control.
[50]						X			Buddy assignment to share game status led to higher engagement which resulted in higher and sustained physical activity levels.

[73]	D					X			Having social connection/friend helped exercise longer; Encouragement from strong partner had negative impact on performance.
[38]						X			Presence of friends related to recording above average step log days by participants.
[92]			X						Greater improvement in food safety among those spending time on Facebook page.
[14]	S				X				The use of private messaging led to an increase in abstinence rates.
[31]			X	X		X			Participants who posted on forum or have social connections, send/receive private messages were more likely to quit smoking.
[32]			X						Reading online forum messages influenced intent to adopt behaviour.
[140]		X						X	Participants with access to virtual world and OSN (QuitNet) components had significant changes in quitting, amount they smoked and increased intentions to quit.
[141]		X		X				X	Participants with access to virtual world and 3D chatroom components were more likely to report abstaining from smoking, smoking fewer cigarettes, quitting smoking.
[55, 56]			X		X	X			Groups with social media features more effective overall.
[117]			X						Using online forums had no effect on outcome (smoking cessation).
